# Application of Ultrasonication as an Emerging Non-Thermal Physical Technology in Meat Product Processing: A Review

**DOI:** 10.3390/foods15101823

**Published:** 2026-05-21

**Authors:** Yun Pan, Chunhua Dai, Lihui Zhang, Man Zhou, Shuyun Zhu, Liurong Huang, Ronghai He

**Affiliations:** 1 School of Food and Biological Engineering, Jiangsu University, Zhenjiang 212013, China; panyun0213@163.com (Y.P.); zhanglh@ujs.edu.cn (L.Z.); manzhou@ujs.edu.cn (M.Z.); shyzhu@ujs.edu.cn (S.Z.); hlr888@ujs.edu.cn (L.H.); heronghai1971@126.com (R.H.); 2 Institute of Food Physical Processing, Jiangsu University, Zhenjiang 212013, China

**Keywords:** ultrasound, meat processing, cavitation, physicochemical properties, microbial inactivation

## Abstract

Meat, as an important source of animal protein, plays a central role in the human diet, and its processing operations critically influence the product quality. As an emerging non-thermal physical technology, ultrasound has demonstrated considerable application potential and distinct advantages in meat processing. This review systematically summarizes recent advances in the application of ultrasound for meat tenderization, marination, sterilization, fermentation, freezing, thawing, drying, and the extraction of bioactive compounds from meat by-products, with particular emphasis on its ability to enhance processing efficiency and final product quality. The underlying mechanisms of ultrasound action in meat systems are discussed in depth. Current evidence indicates that ultrasonication not only intensifies processing operations but also positively modulates the physicochemical and functional properties of meat products, including improved tenderness, water-holding capacity, and color stability, promoted flavor development, reduced cooking loss, and extended shelf life. This review aims to provide a theoretical foundation for the scientific research, practical application, and future development of ultrasound technology in meat processing, highlighting its potential to partially replace conventional methods and contribute to more sustainable food processing practices.

## 1. Introduction

Meat occupies a central position within modern food systems, serving not only as a primary source of high-quality dietary protein but also as a major provider of essential amino acids, B-complex vitamins, and key minerals such as iron and zinc. Owing to its indispensable role in meeting human nutritional requirements, global meat consumption continues to rise, thereby driving the rapid expansion of the meat processing industry [1,2]. However, conventional meat processing operations, such as thermal treatment, curing, drying, freezing, and thawing, are frequently associated with inherent limitations such as low mass transfer efficiency, prolonged processing time, high energy consumption, and undesirable deterioration of nutritional and sensory attributes [3]. For instance, thermal pasteurization may induce excessive protein denaturation and flavor loss [4], traditional marination is time-consuming and often results in heterogeneous salt distribution, while freezing commonly leads to the formation of large ice crystals that cause drip loss and textural damage. Collectively, these challenges constrain further improvements in meat product quality.

In response to increasing demands for environmentally sustainable (green) manufacturing, energy efficiency, and quality upgrading, the development of non-thermal or mild physical processing technologies has emerged as a critical research focus [5]. Over the past decade, a range of emerging technologies, such as cold plasma [6], pulsed electric fields [7], ultrasound [8], ozone [9], pulsed light [10,11], and ultraviolet treatment, have been extensively investigated. These approaches have demonstrated the potential to accelerate processing operations, improve product quality, and mitigate microbial risks while minimizing nutrient degradation [12]. Among them, ultrasound has gained increasing attention due to its ability to induce mechanical and physicochemical effects that enhance food processing efficiency, and it has gradually been adopted in specific food industry sectors [13].

Ultrasound refers to mechanical sound waves with frequencies exceeding the upper limit of human hearing, typically ranging from 20 kHz to 100 MHz. Based on frequency and power intensity, ultrasound is generally categorized into low-intensity high-frequency ultrasound (>1 MHz, <1 W/cm^2^) and high-intensity low-frequency ultrasound (20–100 kHz, 10–1000 W/cm^2^) [14]. In food processing, low-intensity high-frequency ultrasound is primarily employed for analytical purposes, whereas high-intensity low-frequency ultrasound, commonly termed as power ultrasound, is used to modify macromolecular structures and facilitate processes such as foam suppression, emulsification, enzyme inactivation [15] or activation [16,17], and crystallization [18]. The functional effects of power ultrasound are largely attributed to the cavitation effect, which generates localized extreme conditions within the processing medium, including high temperature, pressure, shear forces, and microstreaming. These phenomena collectively induce physical, chemical, and biological modifications, enabling material disruption, enhanced mass transfer, microbial inactivation, and emulsification [19,20,21,22]. Although the application of ultrasound in the food industry has gained momentum only in recent years, particularly in processing fruit juices, jams, soybean products, and soy sauce [23,24,25,26], its advantages, including rapid action, high energy efficiency, low processing temperature, and environmental friendliness, render it especially suitable for addressing bottlenecks in traditional meat processing. Consequently, ultrasound technology has attracted growing interest within the meat industry [27].

In the past decade, accumulating evidence has demonstrated that ultrasound, applied independently or in combination with other processing techniques, can significantly improve meat tenderness and water-holding capacity, inactivate spoilage and pathogenic microorganisms, reduce salt content, enhance cooking yield, and increase the extraction efficiency of valuable components from meat and meat by-products. These findings highlight ultrasound as a versatile platform technology with substantial application potential in meat processing [28,29]. Accordingly, this review aims to systematically summarize and critically evaluate recent advances in ultrasound-assisted meat processing, with particular emphasis on its effects and underlying mechanisms in these procedures. In addition, the impacts of ultrasound on the physicochemical and functional properties of meat products are discussed, and key limitations and challenges associated with current industrial applications are identified. Through an integrated analysis of existing knowledge, this review seeks to provide a theoretical foundation and practical guidance for the rational application and standardized development of ultrasound technology in the meat industry.

## 2. Ultrasound Overview

Ultrasound refers to mechanical waves with frequencies exceeding the upper limit of human hearing (>20 kHz), which propagate through a medium and generate energy capable of inducing physicochemical changes [30]. Ultrasound can be classified into different categories. For example, it is commonly classified into low-intensity (<1 W/cm^2^) and high-intensity (10–1000 W/cm^2^) ultrasound based on its power density. From the perspective of the power–frequency relationship, it can be categorized as high-power low-frequency (20–100 kHz), medium-power intermediate-frequency (100 kHz–1 MHz), and low-power high-frequency (1–100 MHz) ultrasound [31]. Additionally, ultrasound can be differentiated according to frequency modulation (swept versus fixed frequency) and emission mode (pulsed versus continuous operation). As summarized in Table 1.

High-intensity ultrasound (HIU), also termed power ultrasound, has been demonstrated to induce pronounced mechanical, physical, and chemical modifications in food matrices [32,33]. For example, HIU treatment has been reported to enhance the bioactivity, promote amino acid release, and improve protein digestibility in soymilk, while simultaneously reducing antinutritional factors such as phytic acid, trypsin inhibitor, lipoxygenase, β-conglycinin, and glycinin [34]. Consequently, HIU is currently widely applied as an auxiliary technique in enzyme-assisted food processes [35,36]. In contrast, low-intensity and high-frequency ultrasound is primarily employed for non-destructive analytical and diagnostic application, providing valuable information on the physicochemical state, composition, and structural characteristics of food materials [37]. Accumulating evidence indicates that HIU represents a promising technology in meat processing, with the potential to improve the color stability, flavor profile, and tenderness of fresh meat products [38].

In ultrasound processing systems, electrical energy is converted into vibrational (mechanical) energy, which propagates through the processing medium as ultrasonic waves. Based on the elastic properties of the medium and the direction of particle oscillation, these waves can be classified into longitudinal and transverse waves. Longitudinal waves, characterized by particle motion parallel to the direction of wave propagation, transmit energy through alternating cycles of compression and rarefaction. As the predominant ultrasonic wave type, they can propagate efficiently in liquids, solids, and semi-solid food systems [39]. The localized pressure fluctuations, mechanical vibrations, and cavitation effects induced by longitudinal waves are considered key physical drivers of tissue structural modification, mass transfer enhancement, and reaction kinetics acceleration. In contrast, transverse waves are characterized by particle motion perpendicular to the direction of propagation and rely on the shear modulus of the medium for transmission, making them more stable in solid materials. In semi-solid food matrices, the contribution of transverse waves is generally regarded as secondary to that of longitudinal waves. Notably, the propagation velocity of sound waves follows the order of solids > liquids > gases, a principle that underpins the use of ultrasonic pulse techniques for the non-destructive analysis of food composition and structure [40].

**Figure 1:**
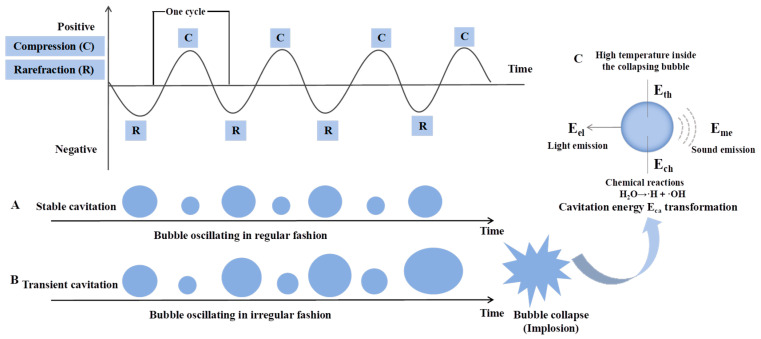
Cavitation effect of ultrasound ((**A**): stable cavitation; (**B**): transient cavitation; (**C**): acoustic energy classification).

Cavitation is the most fundamental and distinctive mechanism underlying the functionality of power ultrasound. It occurs when high-intensity ultrasonic waves propagate through liquid media (e.g., water, saline solutions, or meat tissue fluids) by alternating compression and rarefaction cycles and thus generating periodic positive and negative pressure fluctuations [41]. When the acoustic intensity exceeds a critical threshold, these pressure oscillations induce the nucleation and growth of cavitation bubbles. Based on bubble collapse behavior, cavitation phenomena are typically classified into stable cavitation (Figure 1A) and transient (inertial) cavitation (Figure 1B). During stable cavitation, oscillating bubbles generate microstreaming and localized microjets in the surrounding liquid, while the diffusion of dissolved gases across the bubble–liquid interface further contributes to the formation of localized microcurrents. In contrast, transient cavitation is characterized by the violent collapse of bubbles, resulting in extreme localized physical conditions [42]. As illustrated in Figure 1C, the acoustic energy produced by ultrasonic transducers usually is divided into acoustic propagation energy (Epa) and cavitation energy (Eca). Epa primarily propagates through the medium and is ultimately dissipated as internal energy. Eca, whereas, represents the fraction of acoustic energy absorbed by cavitation bubbles and subsequently converted into multiple energy forms, including mechanical energy (Eme; e.g., cavitation noise, shock waves, and microjets), electromagnetic energy (Eel; e.g., sonoluminescence), thermal energy (Eth; e.g., the extremely high temperatures within collapsing bubbles and heat transfer to the surrounding medium), and chemical energy (Ech; e.g., chemical reactions occurring inside cavitation bubbles) [43]. These events induce intense localized turbulence at the microscale, wherein microjets and shock waves can disrupt cell membranes, fracture polymer chains, and damage biological tissue structures. Moreover, the sonolysis of water molecules generates reactive oxygen species and hydrogen radicals, which may initiate secondary chemical reactions [44].

## 3. Application of Ultrasound in Meat Processing

Owing to its unique physicochemical effects, ultrasound has emerged as a promising technique with considerable application potential across multiple critical stages of meat processing. A growing body of literature has documented its use in various operations, including tenderization, freezing, thawing, cooking, and marination. From a consumer perspective, texture is widely regarded as a primary determinant of meat palatability and overall eating quality. In this context, ultrasound has been shown to exert a pronounced influence on key meat quality attributes, particularly those related to texture [45].

### 3.1. Ultrasound-Assisted Meat Tenderization

Tenderness is commonly recognized as a primary quality attributes of meat products. Conventional tenderization strategies, particularly enzymatic approaches, have long been employed to improve the palatability of low-quality meat cuts. However, plant-derived proteases commonly used in enzymatic tenderization often suffer from poor process controllability, leading to excessive proteolysis, undesirable mushy texture, and quality deterioration due to their non-specific degradation of muscle proteins. Furthermore, the high cost of enzyme purification, structural instability, and activity loss caused by autolysis represent major challenges for industrial applications [46]. Early studies found that meat tenderization could occur as a beneficial by-product during combined thermal and ultrasonic sterilization processes, and ultrasound has since been recognized as an effective alternative or auxiliary technique to conventional tenderization methods [28]. Representative applications of ultrasound in the tenderization of meat and animal products are summarized in Table 2.

To date, numerous studies have proven the feasibility of ultrasound-assisted meat tenderization [53]. For example, ultrasound treatment (500 W, 30 min) reduced the shear force of chicken gizzard by 27.1%, decreased muscle fiber diameter by 26.2%, and increased the myofibrillar fragmentation index (MFI) by 238.1%, demonstrating the effectiveness of appropriate ultrasonic treatment in improving gizzard tenderness [47].

In addition to pork, beef, and lamb, ultrasound has been demonstrated to be effective in tenderizing difficult-to-process muscle foods. For example, whelk (*Buccinum undatum*) meat, which is characterized by high hardness, exhibited a significant reduction in hardness following ultrasound treatment [54]. Jumbo squid (*Dosidicus gigas*) muscle, which is highly sensitive to thermal processing, was effectively softened by ultrasound, accompanied by protein degradation and disruption of muscle fiber structure [55]. In addition to application of ultrasound alone, combination strategies have been explored to enhance tenderization efficacy. The integration of ultrasound with exogenous enzymes has proven effective for beef tenderization [56], and further incorporation of lactic acid was shown to enhance tenderization of yak meat [48]. More recently, ultrasound combined with tumbling or vacuum marination has been reported to significantly accelerate tenderization processes, offering improved processing efficiency and product uniformity [57,58].

#### 3.1.1. Mechanism of Ultrasound-Assisted Meat Tenderization

Meat tenderness is determined by complex and interrelated multiple factors, including intramuscular fat and connective tissue content, sarcomere length, and the activity of proteolytic enzymes [59]. Postmortem degradation of myofibrillar proteins plays a decisive role in this process [60]. Ultrasound-assisted tenderization is widely regarded as a synergistic outcome of multiple mechanisms, primarily including physical disruption of muscle structure, activation of endogenous proteolytic enzymes, enhanced release of calcium ions, and accelerated mass transfer.

**Figure 2:**
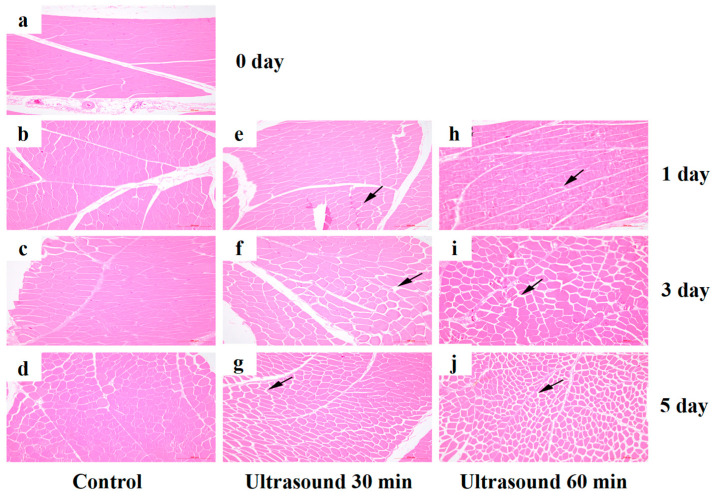
Hematoxylin and eosin staining of muscle fiber bundles in chickens at 1, 3, and 5 days after slaughter ((**a**–**d**): the control; (**e**–**g**): ultrasound 30 min; (**h**–**j**): ultrasound 60 min, respectively). Scale bar = 200 μm [64]. Arrows represented the gap between muscle fibre bundles.

Meat is essentially composed of two major structural components: muscle fibers (which contain the actin-myosin protein system) and intramuscular connective tissue (mainly collagen). Myofibrils are primarily responsible for contraction, whereas connective tissue provides structural support and cohesion. The degree of overlap between actin and myosin filaments governs muscle contraction status and ultimately regulates meat tenderness [61]. Additionally, the content and solubility of collagen, the major component of endomysial connective tissue, directly influences meat toughness [62]. Ultrasound has been shown to exert a pronounced impact on both muscle fibers and connective tissue. In ultrasound-treated beef samples, the mechanical strength of connective tissue decreased significantly. When treatment duration exceeded 10 min, muscle fibers exhibited contraction, endomysial structures were disrupted, perimysial thickness was reduced, and protein aggregation was observed in the extracellular space [63]. Chen et al. [64] systematically investigated the effects of ultrasound on postmortem chicken meat aging and demonstrated that ultrasound accelerated muscle structure degradation by activating apoptosis-related pathways, thereby improving meat tenderness. Morphological observations have revealed that following ultrasonic treatment, the distance between muscle fiber bundles increased (Figure 2), and as treatment time extended, the inter-fiber gaps gradually widen. In goose meat, ultrasound treatment disrupted actin filaments within myofibrils and promoted the transformation of F-actin into G-actin, significantly increasing the myofibrillar fragmentation index and inducing myofibrillar disintegration. Consequently, these findings indicate that ultrasound facilitated meat tenderization through mechanical disruption of myofibrillar architecture and connective tissue, coupled with biochemical changes that enhance proteolytic degradation [65].

In summary, the mechanisms underlying ultrasound-assisted meat tenderization can be categorized into four interrelated physical and biochemical processes. First, during ultrasound propagation, differential displacement and acceleration of ions within the medium generate intense mechanical vibrations, while cavitation-associated physical forces disrupt weak intermolecular interactions and induce structural modifications in muscle tissue. Second, ultrasound promotes the release of lysosomal proteases and increases intracellular Ca^2+^ concentration [66], thereby activating the calpain system and accelerating protein hydrolysis. Third, the so-called “sponge effect” induced by ultrasound enhances marinade penetration, further facilitating tenderization. Finally, the extreme localized conditions generated during cavitation bubble collapse, including high pressure, shear forces, and microjets, directly contribute to myofibrillar protein degradation and collagen loosening, resulting in a marked improvement in meat tenderness [67].

#### 3.1.2. Effects of Ultrasound-Assisted Tenderization on Meat Quality

Beyond its primary role in improving tenderness, ultrasound-assisted tenderization exerts multidimensional effects on the textural, functional, and sensory properties of meat products. From a textural perspective, ultrasound treatment has been reported to enhance water-holding capacity (WHC) [68]. Changes in WHC are closely associated with postmortem alterations in myofibrillar structure, highlighting the intrinsic link between tenderness development and water distribution during the conversion of muscle to meat.

Regarding sensory attributes, ultrasonication has been shown to improve color stability and promote flavor development. Color serves as the primary visual attribute influencing consumer purchase decisions and is predominantly determined by the redox state of myoglobin. Specifically, oxymyoglobin and carboxymyoglobin impart meat bright cherry-red and red hues, respectively. Deoxymyoglobin appears purplish-red, while metmyoglobin is associated with undesirable brown discoloration. Ultrasound-induced structural modifications may affect myoglobin chemical states, thereby influencing meat color stability.

From a processing functionality perspective, ultrasound-assisted tenderization has been shown to reduce cooking loss and improve WHC. For instance, the combination of ultrasound with enzymes and lactic acid for yak meat tenderization reduced cooking loss by 31.25% and significantly improved WHC compared with untreated samples [48]. Similarly, increasing ultrasound power and treatment duration was shown to enhance the WHC of bovine myofibrillar proteins [69]. For chicken breast meat, ultrasound combined with a low-concentration sodium bicarbonate solution not only improved tenderness but also reduced filtration residue, cooking loss, and shear force [70]. Furthermore, ultrasound-assisted tumbling effectively enhanced both the tenderness and WHC of woody chicken breast, enabling moderately treated woody meat to achieve quality levels comparable to untreated normal chicken breast [57]. These findings are consistent with those reported by Roobab et al. [71], who observed that ultrasound-assisted tenderization reduced pH and cooking loss while increasing lightness and yellowness values in chicken breast meat.

### 3.2. Ultrasound-Assisted Meat Cooking

Cooking methods exert a significant influence on the quality of meat products. Conventional thermal processing often leads to the deterioration of nutritional value, flavor, and color. In response, increasing attention has been paid to non-thermal or mild processing technologies that better preserve these quality attributes. Among them, ultrasound has emerged as a promising auxiliary technique due to its ability to enhance heat and mass transfer, a critical requirement during meat cooking.

Previous studies have demonstrated that ultrasound-assisted cooking can achieve textural and flavor characteristics comparable to or even superior to those obtained by traditional braising, while operating at lower temperatures or shorter processing times, thereby contributing to energy savings [72]. Under ultrasonic conditions, faster cooking rates, higher water retention, and reduced cooking losses have been consistently reported [73]. Moreover, cooked meat treated with ultrasound exhibits improved tenderness, characterized by increased muscle fiber diameter and a greater degree of fiber disruption compared to meat cooked by conventional convective heating alone.

Appropriate ultrasonic power levels (e.g., 450 W) have been shown to simultaneously improve the physicochemical properties, microstructure, and sensory quality of pork meatballs, resulting in an increase in cooking yield from 82.55% to 92.87% [74]. Beyond improvements in processing efficiency and cooked flavor, ultrasound-assisted braising has also been reported to extend the shelf life of meat products [75]. In recent years, the application of ultrasound in food frying has attracted considerable research interest, as it significantly enhances frying efficiency, product quality, and processing safety [76]. Notably, ultrasound-assisted frying contributes to a reduction in oil uptake, thereby improving the safety and nutritional profile of fried foods [76,77].

In practical applications, ultrasound is frequently combined with other emerging processing technologies, such as sous-vide cooking [78] and pulsed electric field treatment [79], to further enhance the quality of meat products.

#### 3.2.1. Mechanism of Ultrasound-Assisted Meat Cooking

The benefits of ultrasound-assisted meat cooking are principally due to the synergistic impact of cavitation, mechanical vibration, and concomitant thermal effects. During ultrasonic propagation in the cooking media, numerous cavitation bubbles are generated. The expansion and subsequent collapse of these bubbles cause localized high temperatures and pressures, and shock waves, which physically disrupt muscle fibers and connective tissues. As a result, the inter-fiber spaces are enlarged, forming microchannels that facilitate water redistribution and retention within the muscle matrix, while simultaneously enhancing heat and mass transfer efficiency.

In addition, ultrasonic vibration disrupts the stagnant boundary layer on the meat surface, thereby accelerating the penetration of seasonings and flavor compounds into the tissue and enhancing flavor development. The microjets and localized thermal effects produced during cavitation bubble collapse further contribute to accelerated cooking kinetics. Yang et al. [80] reported that low-temperature braised rabbit legs treated with ultrasound at different frequencies exhibited pronounced muscle fiber contraction and structural damage, confirming the disruptive effect of ultrasound on meat microstructure.

Furthermore, ultrasound treatment has been demonstrated to alter the conformation and surface properties of myofibrillar proteins, which are closely associated with their enhanced binding capacity for specific aroma and flavor compounds [81]. Overall, the synergistic effects of cavitation, microjet formation, and thermal phenomena acting on proteins and other macromolecular components constitute the fundamental mechanisms by which ultrasound improves texture, sensory attributes, and processing efficiency during meat cooking [82].

#### 3.2.2. Effect of Ultrasound-Assisted Meat Cooking on the Quality of Meat Products

Extensive evidence indicates that ultrasound-assisted cooking exerts beneficial effects on meat quality, particularly by reducing cooking loss, improving WHC, and increasing product yield. Wang et al. [78] systematically investigated the effects of ultrasound treatment (28 kHz, 60 W, 71 °C for 37 min) combined with sous-vide cooking (71 °C for 40–120 min) on the textural properties, water distribution, and protein characteristics of braised beef. The results demonstrated that ultrasound-assisted sous-vide cooking within 100 min significantly improved both texture quality and water retention.

The synergistic application of ultrasound and pulsed electric field (PEF) technology has also yielded promising results. Chicken breast meat treated with ultrasound (24.5 kHz, 300 W, 10 min) followed by PEF at different electric field intensities (1.6, 3.3, and 5.0 kV/cm for 30 s) exhibited a significant reduction in cooking loss, with a maximum decrease of 28.78%, leading to a marked improvement in cooking yield, while no significant adverse effects on overall color were observed [79].

Beyond textural and yield-related attributes, ultrasound-assisted cooking has been shown to promote flavor formation in meat products [83]. Meat flavor is composed of aroma and taste components. Aroma compounds are mainly derived from lipid oxidation and Maillard reactions [84], whereas taste-active substances primarily include proteins, peptides, free amino acids, nucleotides, and reducing sugars [85]. Studies have revealed that ultrasound-assisted cooking of braised beef not only enhances its nutritional value and taste complexity but also significantly increases both the diversity and relative content of volatile flavor compounds (*p* < 0.05), particularly aldehydes, alcohols, and ketones [86].

In addition to its effects on texture and sensory quality, ultrasound-assisted braising has been reported to inhibit microbial growth and delay lipid oxidation and protein deterioration, thereby effectively extending the shelf life of spiced cooked beef products [75].

### 3.3. Ultrasound-Assisted Meat Brining

Meat brining is a traditional food preservation technique widely applied in meat processing. During this process, meat is immersed in a saturated salt solution, allowing it to absorb additional water and salt. The salt concentration in the brine surrounding muscle tissue is higher than that inside the cells, which drives salt ions to diffuse into the muscle tissue due to the concentration gradient [87]. However, the migration of sodium chloride from the brine into the muscle matrix is generally slow and often results in heterogeneous salt distribution, which negatively affects product uniformity and quality. In addition, adding large amounts of salt (NaCl) during the curing process can have adverse effects on consumer health; therefore, efforts are being stepped up to reduce the salt content in processed foods [88].

Ultrasound-assisted brining has been demonstrated to significantly shorten brining time while markedly improving the uniformity of salt and nitrite distribution within meat products [89]. Studies have shown that ultrasound treatment at various intensities enhances NaCl diffusion, with tri-frequency simultaneous ultrasound at an intensity of 101.3 W L^−1^ being particularly effective in accelerating NaCl transport and achieving a more homogeneous salt distribution [90]. Furthermore, Guo et al. [91] reported that ultrasound treatment enhanced the WHC of pork while increasing the NaCl content of the final product to approximately 1%. This increase was attributed to ultrasound-induced cavitation, which enlarged the interstitial gaps between muscle fibers and promoted NaCl diffusion, as confirmed by scale-up tests in a domestic refrigerator.

The combined application of ultrasound and microbubble treatment has also been shown to effectively enhance salt diffusion, alter muscle microstructure, and influence the thermal stability of proteins [92]. These effects not only improve processing efficiency but also provide promising opportunities for the development of low-sodium meat products. A more uniform salt distribution allows for reduced salt usage while maintaining equivalent saltiness perception and preservative efficacy, without inducing negative effects on protein or lipid oxidation [93].

#### 3.3.1. Mechanism of Ultrasound-Assisted Meat Brining

The enhancement of meat brining by ultrasound is primarily attributed to its cavitation and acoustic streaming effects. The cavitation effect generated during ultrasonic treatment creates microscopic, irreversible pores and channels on the surface and within muscle tissues, providing new pathways for the diffusion of curing agents. In addition, microstreaming disrupts muscle fiber membranes and the endomysium, thereby reducing cellular barriers to mass transfer [94].

Simultaneously, acoustic streaming induces vigorous agitation of the stagnant liquid layer surrounding meat samples and promotes bulk movement of the brine, substantially intensifying the diffusion process driven by concentration gradients, as described by Fick’s second law. Pan et al. [95] investigated the microstructural changes in pork biceps femoris muscle subjected to static and ultrasound-assisted brining (350 W for 1 h). Compared with static brining alone, samples treated with ultrasound-assisted brining exhibited more compactly arranged muscle fibers and an increased number of microcracks, indicating enhanced salt diffusion. Moreover, SDS-PAGE analysis revealed significant loss of major myofibrillar protein components following ultrasound-assisted brining.

Ultrasound intensity has been shown to exert a significant influence on the diffusion coefficients of both NaCl and water, with diffusion coefficients increasing markedly as ultrasound intensity rises. Transmission electron microscopy further confirmed that the inter-myofibrillar spacing expanded with increasing ultrasonic intensity [96]. However, the efficacy of ultrasound in meat brining is highly dependent on processing parameters. Inguglia et al. [97] demonstrated that the geometric characteristics of ultrasonic systems, including probe size and probe-to-sample distance, play a critical role in salt uptake efficiency and are key determinants of brining performance.

#### 3.3.2. Effect of Ultrasound-Assisted Brining on the Quality of Meat Products

**Figure 3:**
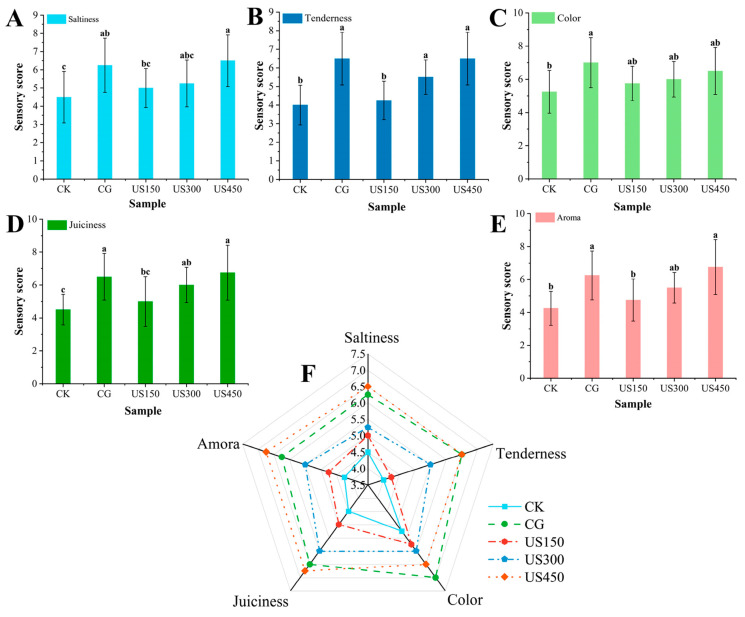
Sensory evaluation scores and salt (NaCl) content of braised duck under different ultrasonic conditions (CK: control group; CG: curing group; US150, US300, and US450: ultrasound-assisted curing at 150, 300, and 450 W, respectively) (**A**–**E**): attributes assessment of saltiness, tenderness, color, juiciness, and aroma, respectively; and (**F**): a radar chart comparing overall sensory attributes). Different letters denote significant differences among treatments (*p* < 0.05).

Ultrasound-assisted brining not only enhances curing efficiency but also significantly affects the quality attributes of meat products, such as WHC, color, and textural properties [95]. For example, ultrasound-assisted brining has been shown to promote salt penetration in sea bass, improve texture, and enhance water retention, while simultaneously enriching flavor by promoting protein degradation and lipid oxidation. Under the optimal conditions, ultrasonic treatment significantly increased the total free amino acid content [98]. Wang et al. [99] investigated the effects of ultrasound-assisted curing on salt penetration in sauced duck. Sensory evaluation scores of marinated duck under different ultrasound treatments are presented in Figure 3A–F. Compared with the low-power treatment groups (US150 and US300, with scores of 5.00 ± 1.06 and 5.25 ± 1.28, respectively) and the control group (CK, 4.50 ± 1.41), high-power ultrasound treatment (US450, 6.50 ± 1.41) significantly enhanced the saltiness of the marinated duck (*p* < 0.05) (Figure 3A). Sensory evaluation indicated a slight improvement in tenderness perception (Figure 3B). Although variations in juiciness were observed across different ultrasound power levels (Figure 3D), ultrasound treatment had no significant impact on flavor or color attributes. The overall influence of ultrasound on the quality of marinated duck is comprehensively illustrated in the radar chart (Figure 3F).

Beyond physicochemical quality attributes, high-intensity and long-duration ultrasound treatment has been reported to effectively inactivate pathogenic microorganisms in brining systems, thereby extending the shelf life of cured products. This antimicrobial effect is attributed to the synergistic action of physical disruption of microbial cell membranes and chemically induced oxidative damage [100]. The underlying mechanisms of ultrasound-induced microbial inactivation will be discussed in detail in the following section.

### 3.4. Antimicrobial Action of Ultrasound in Meat Processing

Meat products are rich in nutrients and water, which provide a favorable environment for the growth of pathogenic and spoilage bacteria [101]. Common microorganisms associated with meat contamination include *Staphylococcus aureus* [102], *Escherichia coli* O157:H7 [103], *Pseudomonas* spp. [104], *Listeria monocytogenes* [105], and *Bacillus cereus* [106]. Conventional microbial inactivation strategies in meat processing rely primarily on thermal treatments. However, extensive evidence has demonstrated that high-temperature processing adversely affects the functional and sensory properties of foods, particularly thermolabile components.

As a non-thermal physical processing technology, ultrasound has long been investigated for microbial inactivation and has demonstrated considerable efficacy. For example, ultrasonication achieved a reduction of up to 99.999% in enterohemorrhagic *E. coli* O157:H7 biofilms formed on polystyrene surfaces [107]. In recent years, increasing attention has been paid to the application of ultrasound for ensuring the microbial safety of meat products, owing to its promising antimicrobial potential [108]. Representative applications of the antimicrobial action of ultrasound in meat systems are summarized in Table 3.

For instance, ultrasonication (35 kHz, 2 min) of cooked smoked lamb inhibited the growth of *Candida albicans*, *E. coli*, *Bacillus subtilis*, and *S. aureus* by 33.3%, 43.8%, 46.8%, and 80.6%, respectively, compared to untreated samples. While applied at 26 kHz for 1 min, ultrasound achieved reduction of 50.0%, 64.6%, 89.1%, and 86.8%, respectively, for the same microorganisms. Prolonged treatment further increased bacterial inhibition rates to 90–98% [110]. High-intensity ultrasound (40 kHz, 9.6 W/cm^2^) applied for 50 min has also been proposed as an effective control strategy for *S. aureus* in chicken breast meat [108].

In practical applications, ultrasound is frequently integrated with other antimicrobial approaches to form hurdle technologies, thereby enhancing microbial inactivation efficiency and penetration in complex meat matrices [117]. The most common strategy involves the combination of ultrasound with chemical disinfectants or natural antimicrobial agents [22,118]. For example, synergistic inactivation of *S. aureus* and *Salmonella enterica* has been observed when ultrasound is combined with phenyllactic acid in bacterial suspensions [119]. In meat processing systems, ultrasound combined with sodium hypochlorite effectively reduced total viable counts and *Enterobacteriaceae* on chicken leg-quarters [116]. Moreover, the combination of ultrasound (130 kHz) with cetylpyridinium chloride (0.1%) or sodium hypochlorite (0.01%), together with vacuum packaging, reduced *Campylobacter* counts on broiler carcasses [120]. Another study reported that ultrasound alone (37 kHz, 380 W, 5 min) was ineffective against *Salmonella Typhimurium* and *Campylobacter jejuni* on chicken skin, but peroxyacetic acid (50–200 ppm) was effective [121]. These findings highlight the potential of hurdle technologies that combine ultrasound with chemical sanitizers to ensure the microbial safety of meat products.

Beyond chemical-assisted approaches, combined physical technologies, such as manosonication (pressure–ultrasound), thermosonication (ultrasound–heat), and manothermosonication (pressure–heat–ultrasound), have been identified as highly effective microbial inactivation methods [122,123]. Recent studies suggest that combining ultrasound with other non-thermal technologies, such as high hydrostatic pressure or pulsed electric fields, can achieve more comprehensive microbial control through multi-target mechanisms. These emerging technologies demonstrate strong potential to complement or partially replace conventional ultrasound–chemical treatment strategies currently used in the food preservation industry [124].

#### 3.4.1. Mechanism of the Antimicrobial Action of Ultrasound in Meat

**Figure 4:**
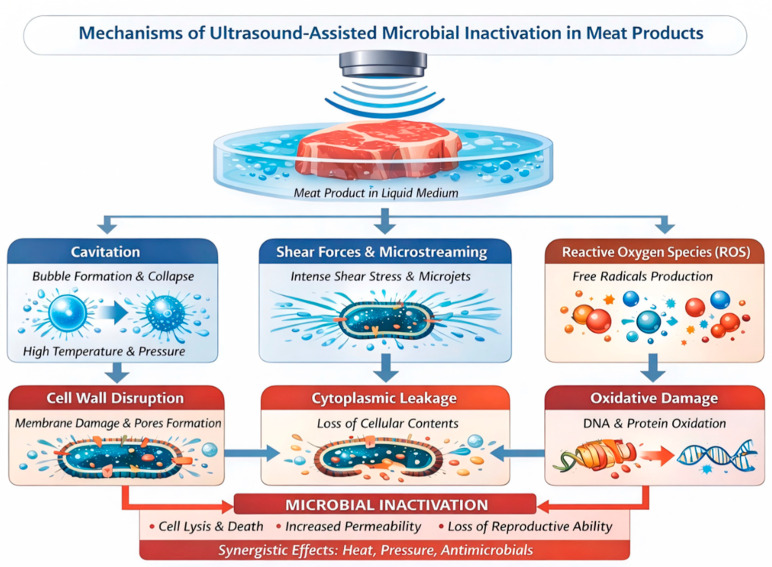
Mechanism of the antimicrobial action of ultrasound on meat products.

**Figure 5:**
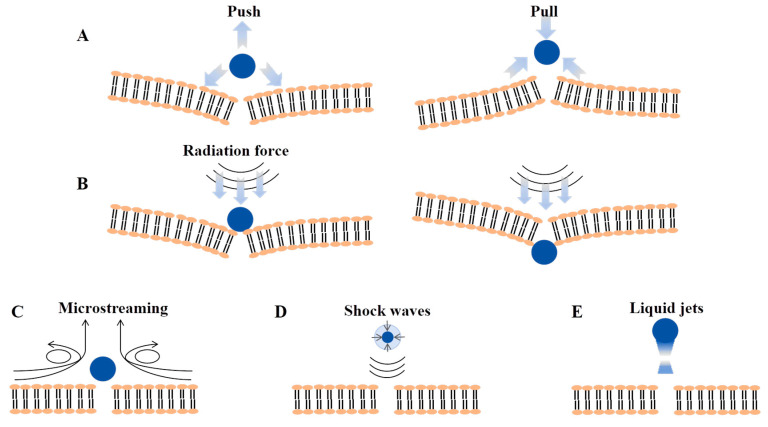
Schematic diagram of cavitation-induced cell membrane perforation mechanisms (**A**): stable bubble oscillation near the cell surface generates attractive and repulsive forces, disrupting membrane integrity; (**B**): ultrasonic radiation force converted into bubble kinetic energy, enabling compressed bubbles to penetrate the cell membrane and enter the cell; (**C**): stable oscillation of attached cavitation bubbles producing microstreaming and shear forces to rupture the membrane; (**D**): shockwaves from cavitation bubble collapse exerting an impulsive force, causing membrane perforation; (**E**): asymmetric bubble collapse producing liquid jets.

The antimicrobial efficacy of ultrasound primarily originates from physical, chemical, and biological effects induced by acoustic cavitation [125]. The mechanism of the antimicrobial action of ultrasound is illustrated in Figure 4. In meat systems, the collapse of cavitation bubbles generates localized high temperatures, high pressures, intense shear forces, and microjets, which can directly disrupt microbial cells attached to the meat surface or present in superficial tissues, leading to membrane perforation and leakage of intracellular contents [126]. In addition, cavitation induces the formation of reactive oxygen species (ROS), which oxidize critical cellular components such as membrane lipids, enzymatic proteins, and nucleic acids, thereby inhibiting or inactivating spoilage and pathogenic microorganisms [127]. The phenomenon of ultrasonic perforation of cell membranes is termed the sonophoretic effect, the mechanism of which is illustrated in Figure 5 [127]. During sterilization with antimicrobial adjuvants, this perforation effect increases the permeability of bacterial membranes, allowing antimicrobial agents to enter bacterial cells through the formed channels. This subsequently disrupts proteins, enzymes, and DNA, and may even lead to the leakage of intracellular substances.

The antimicrobial effectiveness of ultrasound is strongly influenced by multiple factors, including microbial species, food matrix composition, microbial load, treatment temperature, and ultrasonic parameters. Notably, significant differences have been observed between Gram-positive and Gram-negative bacteria. Gram-positive bacteria generally exhibit greater resistance to ultrasound due to their thicker peptidoglycan cell walls [128]. Similarly, spore-forming bacteria are more resistant to ultrasound than vegetative cells and also display higher tolerance to thermal treatments. Moreover, the efficiency of ultrasound is directly proportional to its intensity and frequency, with higher energy inputs generally resulting in greater microbial inactivation [115].

#### 3.4.2. Effect of the Antimicrobial Action of Ultrasound on the Quality of Meat Products

In addition to its antimicrobial effects, ultrasound treatment has been shown to better preserve the color, texture, nutrition, and flavor compounds of meat products than traditional thermal sterilization procedures [129]. Krasnikova et al. [110] evaluated the sensory properties of cooked smoked lamb subjected to ultrasound-assisted brining for 6, 8, 10, and 12 h, and found that samples ultrasonicated for 12 h exhibited the most favorable sensory quality. Similarly, Owusu-Ansah et al. [112] reported that ultrasound and multifrequency thermosonication treatments improved pH, texture, and color attributes of pork, as well as achieved efficient microbial inactivation and maintained or enhanced overall product quality.

More recently, in-package ultrasound treatment has emerged as an efficient and energy-saving preservation strategy for meat products. Compared with conventional unpackaged ultrasound treatment, in-package ultrasonication using low-density polyethylene packaging showed superior preservation effects. In frozen chicken breast, peroxide value and free fatty acid levels were reduced by 15.0% and 17.6%, respectively, while total viable counts decreased by 37.4%, and tenderness was significantly improved [130].

Valenzuela et al. [111] investigated the effects of HIU (40 kHz, 11 W/cm^2^) on the physicochemical properties and shelf life of beef semitendinosus muscle stored at 4 °C for 0, 3, 6, and 9 days. They found that HIU treatment significantly improved storage quality, maintained lowered pH values compared with untreated samples, and markedly slowed overall color changes, with the total color difference (ΔE) decreasing from 5.99 to 1.43. Similarly, numerous studies have shown that ultrasound-assisted sterilization can effectively extend the shelf life of meat products under standard refrigerated or frozen storage conditions by inhibiting microbial growth and delaying oxidative deterioration [130,131,132].

### 3.5. Ultrasound-Assisted Meat Fermentation

Although ultrasound has been widely applied for microbial inactivation and food sterilization, its biological effects are highly dependent on processing conditions, such as treatment time, power density, frequency, and medium characteristics [133]. Ultrasonication, at low frequencies (approximately 70 kHz) and low acoustic intensities (<2 W/cm^2^), has been shown to enhance the growth of *S. aureus*, *P. aeruginosa*, and *E. coli* compared to without ultrasound treatment. In contrast, higher acoustic intensities can cause bacterial cells to detach from polyethylene surfaces [134].

In recent years, increasing attention has been paid to the use of moderate or sublethal ultrasound intensities to stimulate the activity of beneficial microorganisms, such as *Lactobacillus* and *Bifidobacterium* spp. [135,136]. Although research on ultrasound-assisted fermentation in meat products remains limited, available studies suggest substantial application potential to accelerate fermentation kinetics, enhance flavor development, and regulate microbial community dynamics [137]. Italian salami, a traditional dry-fermented sausage, has been used as a representative model system. Ultrasound treatment, particularly for 9 min, significantly promoted the growth of lactic acid bacteria and members of the *Micrococcaceae* family during fermentation (*p* < 0.05) [138]. Moreover, ultrasound can be combined with functional fermentation media, such as acid whey, to improve processing efficiency while maintaining the quality of fermented meat products [139].

Freshly prepared fermented foods often exhibit undesirable sensory attributes, including harsh texture, pungent odors, and unattractive color. The maturation stage is therefore critical for achieving product stability and quality. Ultrasonic irradiation has been shown to significantly accelerate this process, thereby improving these sensory attributes of fermented foods [140].

#### 3.5.1. Mechanism of Ultrasound-Assisted Meat Fermentation

**Figure 6:**
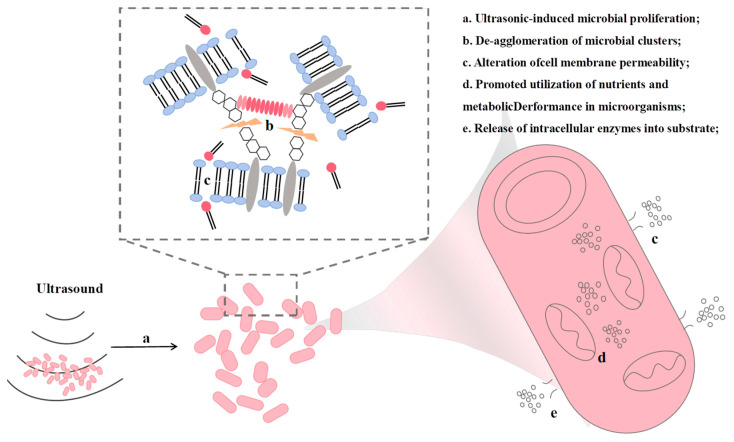
Mechanism of ultrasonic stimulation on microorganisms.

Ultrasound-assisted fermentation is driven by the synergistic effects of acoustic cavitation and mechanical vibration, which operate across multiple scales to enhance fermentation processes. At the cellular level, ultrasound increases microbial cell membrane permeability, facilitating nutrient uptake and the release of metabolic products, accelerating the proliferation and shortening the fermentation lag phase. The mechanism by which ultrasound promotes microbial proliferation is shown in Figure 6 [141].

At the molecular level, ultrasound alters enzyme conformations, thereby reducing activation energy barriers and enhancing catalytic efficiency. Simultaneously, ultrasound disrupts substrate structures, exposing additional enzymatic binding sites and significantly increasing enzymatic hydrolysis rates and metabolic flux. At the system level, localized extreme conditions generated by ultrasonic cavitation promote key maturation-related reactions, including Maillard reactions, esterification, and protein hydrolysis, which collectively contribute to the formation of flavor compounds and bioactive compounds.

#### 3.5.2. Effect of Ultrasound-Assisted Fermentation on the Quality of Meat Products

Beyond its effects on fermentation kinetics, ultrasound treatment has been shown to significantly improve the texture and overall quality of fermented meat products, promote flavor formation, and reduce off-flavors. For instance, a combined strategy involving ultrasound pretreatment of duck liver and ultrasound-stressed yeast fermentation significantly increased ω-3 fatty acid content, improved textural stability, reduced water activity, and lowered thiobarbituric acid reactive substance values, thereby alleviating undesirable odor perception in fermented duck liver [142]. Similarly, ultrasound pretreatment followed by fermentation with *Pediococcus pentosaceus* markedly enhanced protein hydrolysis in chicken liver. The amino nitrogen content increased from 2.99 ± 0.18 g/100 g to 7.70 ± 0.11 g/100 g, while total free amino acids and hydrolyzed amino acids increased by 39.38% and 41.77%, respectively. Notably, the contents of essential amino acids and umami-related amino acids were significantly elevated, accompanied by a reduction in bitterness and an enhancement of sourness [143]. These findings are consistent with previous observations reported by Hu et al. [144].

The treatment duration of ultrasonication in dry-fermented sausages has been shown to significantly influence biochemical processes during fermentation. Moderate ultrasound exposure (3–6 min) effectively promoted protein hydrolysis and lipid oxidation, leading to increased levels of flavor precursors and volatile aroma compounds [145]. Regarding color attributes, ultrasound may influence long-term color stability during maturation by promoting oxidative reactions, thereby improving the color of fermented beef products [139]. However, it should be noted that ultrasound treatment may exert adverse effects under certain conditions. For example, although ultrasound negatively affected color, odor, and taste attributes of fermented dry-cured yak meat, it significantly improved tenderness and overall acceptability [146].

Collectively, these findings indicate that ultrasound treatment has the potential to simultaneously improve fermentation efficiency and enhance sensory attributes, such as flavor, texture, mouthfeel, and color of fermented meat products [147], providing an effective physical strategy for process innovation in traditional fermented foods [148].

### 3.6. Ultrasound-Assisted Meat Freezing

Freezing is widely applied in meat preservation due to its ability to inhibit microbial growth and physicochemical and biochemical reactions associated with food deterioration, can significantly extend the shelf life of meat and meat products. However, during slow freezing, large, irregularly shaped ice crystals with sharp edges tend to form in the extracellular space, which can severely damage muscle cells and tissue structures. This damage often results in increased drip loss upon thawing and a consequent decline in product quality [149].

Ultrasound-assisted freezing has emerged as a highly promising application of ultrasound technology in recent years. Accumulating evidence indicates that ultrasound can significantly accelerate the freezing process and effectively mitigate quality deterioration associated with conventional freezing techniques [150]. Representative applications of ultrasound-assisted freezing are summarized in Table 4.

In addition to accelerating freezing, ultrasound-assisted frozen meat products exhibit reduced thawing loss and cooking loss during subsequent processing [156]. For instance, ultrasound-assisted immersion freezing (UIF) at appropriate power levels significantly accelerated the freezing rate of pork *longissimus dorsi* muscle and effectively reduced the migration and loss of immobilized and free water, thereby decreasing thawing loss [157]. Multiple studies have consistently demonstrated that UIF is an effective freezing strategy for suppressing quality deterioration in meat products during frozen storage [156].

Beyond immersion-based systems, Astráin-Redín et al. [151] evaluated the application of ultrasound in a direct-contact freezing system without liquid-mediated energy transfer. Chicken breast samples were frozen in a forced-air cooling tunnel operating between −3 and −2 °C. The results showed that ultrasound treatment shortened freezing stages, reducing total freezing time by approximately 11%. These findings indicate that ultrasound has considerable potential for accelerating food freezing processes in both liquid-mediated and non-liquid-mediated environments.

#### 3.6.1. Mechanism of Ultrasound-Assisted Meat Freezing

The enhancement of freezing efficiency by ultrasound is primarily attributed to acoustic cavitation effects. Studies have demonstrated that ultrasound-assisted freezing effectively reduces the size of ice crystals formed during freezing [157]. For example, multifrequency ultrasound-assisted immersion freezing significantly alleviated mechanical damage and protein denaturation in *Macrobrachium rosenbergii* muscle, accompanied by producing smaller and more uniformly distributed ice crystals [153]. Similarly, ultrasound-assisted immersion freezing of perch fillets resulted in more favorable ice crystal morphology [155].

During freezing, ultrasound-induced cavitation serves as an effective nucleation catalyst, triggering the rapid formation of numerous uniformly distributed ice nuclei. The shock waves generated by cavitation bubble collapse can fragment existing ice crystals, thereby creating additional nucleation sites. This cascade promotes the formation of numerous small ice crystals, substantially reducing mechanical damage and preserving the structural integrity of muscle cells [158]. Furthermore, microjets induced by cavitation disrupt the solid–liquid interfacial boundary layer, significantly enhancing heat and mass transfer rates. The combined action of cavitation and microjets reduces thermal resistance and shortens freezing time, effectively suppressing the growth of large ice crystals. The resulting smaller ice crystals minimize structural damage to muscle tissues, thereby maintaining the texture, WHC, and nutritional quality of frozen meat products [159].

#### 3.6.2. Effect of Ultrasound-Assisted Freezing on the Quality of Meat Products

The application of ultrasound during freezing contributes to the preservation of multiple physicochemical properties of meat products. Improved cellular integrity results in enhanced tenderness, WHC, and higher product yield. Smaller ice crystals cause less damage to muscle pigment proteins, such as myoglobin, allowing thawed meat to better retain a fresh red appearance and preventing discoloration.

For example, there were no significant differences (*p* > 0.05) between ultrasound-assisted frozen pork *longissimus dorsi* muscle and fresh meat in terms of color parameters (redness a*, yellowness b*), pH, and cooking loss [157]. Similarly, UIF more effectively preserved the WHC, texture, microstructure, and protein stability of perch fillets, resulting in significantly superior frozen storage quality than conventional rapid freezing at the same temperature [155]. Ultrasound treatment has also been proven to be more effectively in inhibiting lipid oxidation during frozen storage than non-ultrasound treatments [160]. Notably, ultrasound-assisted freezing did not adversely affect the digestibility of chicken proteins [151]. Collectively, these findings demonstrate that ultrasound-assisted freezing effectively preserves physicochemical properties and prevents quality deterioration in meat products throughout frozen storage.

However, the effects of ultrasound on meat quality vary depending on whether it is applied before or after freezing [161]. HIU applied prior to freezing resulted in brighter and more vivid orange-yellow coloration, whereas post-freezing HIU treatment shifted meat color toward a lighter red tone. Pre-freezing HIU accelerated the resolution of rigor mortis and significantly reduced pH immediately after treatment. This pH decline can be attributed to cavitation-induced disruption of muscle cell membranes, which releases intracellular acidic contents, and accelerated post-mortem glycolysis leading to lactic acid accumulation [64,66]. In contrast, prolonged post-freezing HIU treatment (40 min) significantly increased weight loss and induced meat toughening, whereas short-duration post-freezing HIU alleviated freezing-induced quality deterioration and significantly improved WHC during subsequent refrigerated storage. Importantly, pre-freezing HIU treatment had no significant effect on the yield (weight loss) of rabbit meat. These results indicate that pre-freezing application of HIU represents a promising strategy for improving tenderness and WHC of meat products.

### 3.7. Ultrasound-Assisted Meat Thawing

During thawing, a series of physicochemical changes occur that critically affect key quality attributes of meat products, including flavor, texture, and color. Conventional thawing procedures are often associated with slow thawing rates, intensified lipid oxidation, protein structural damage, and excessive drip loss, all of which deteriorate product quality [162]. The application of ultrasound in meat thawing has been investigated for decades. Low-intensity ultrasound has been successfully employed as a non-destructive and intelligent monitoring tool, enabling real-time and high-accuracy prediction of thawing status in beef and thereby improving thawing quality and safety [163]. In contrast, HIU has recently emerged as a highly promising strategy for accelerating thawing processes.

HIU represents an efficient and high-quality thawing technique. Representative applications of ultrasound-assisted thawing of frozen foods are summarized in Table 5. Ultrasound-assisted thawing has been proven to markedly shorten thawing time and reduce thawing loss [164,165]. For example, compared with air-thawed samples, ultrasound-assisted immersion thawing (UIT) of chicken breast at 300 W shortened thawing time by 57%, while minimizing damage to myofibrillar protein structures [166].

Despite its high efficiency, ultrasound-assisted thawing has several inherent limitations, including restricted penetration depth, relatively high energy consumption, and the risk of localized overheating. When ultrasound propagates through frozen food matrices, a large percentage of acoustic energy is dissipated and converted into heat. Consequently, the surface temperature of frozen foods increases more rapidly than the internal temperature, potentially causing surface overheating and quality deterioration. As a result, extensive efforts have been devoted to identifying optimal ultrasound-assisted thawing conditions. To overcome the drawbacks of ultrasound-only treatments, ultrasound-assisted thawing is frequently combined with other technologies, such as ultrasound-assisted far-infrared thawing and ultrasound-assisted microwave thawing [170].

#### 3.7.1. Mechanism of Ultrasound-Assisted Meat Thawing

The enhancement of thawing efficiency by ultrasound is primarily attributed to acoustic cavitation and microstreaming, which enhance heat transfer between the external water bath and the interior of meat samples, thereby shortening thawing time and reducing drip loss and quality deterioration, while better preserving meat color and texture [171]. Furthermore, ultrasound-induced microdisturbances promote rapid melting of ice crystals and facilitate more uniform redistribution of intramuscular water, preventing localized overheating and excessive protein denaturation [172]. The migration rate of the freezing–thawing interface is closely related to ultrasound intensity, allowing ultrasound-assisted thawing to accelerate the process while minimizing structural damage. However, ultrasonic cavitation-induced surface overheating and the limited penetration depth, particularly at higher frequencies due to increased attenuation, may adversely affect product quality. Therefore, careful optimization of ultrasonic parameters is essential to balance thawing acceleration and quality preservation [173].

#### 3.7.2. Effect of Ultrasound-Assisted Thawing on the Quality of Meat Products

**Figure 7:**
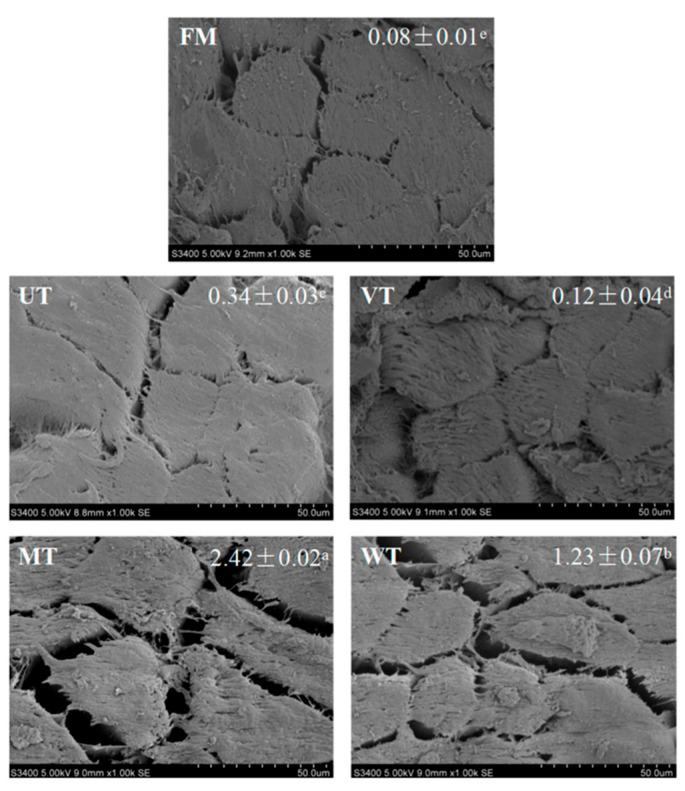
Microstructure of porcine *longissimus dorsi* thawed by different methods (FM: fresh meat; UT: ultrasound thawing (20 °C); VT: vacuum thawing (25 °C); MT: microwave thawing; WT: water immersion thawing (14 °C)). The gap areas (mm^2^) are given as the means ± SD, with different lowercase letters (a–e) indicating significant differences (*p* < 0.05) [175].

Numerous studies have investigated the effects of ultrasound-assisted thawing on the quality of thawed foods. Overall, this technology has been shown to improve WHC, preserve meat quality, and reduce structural damage during thawing [174]. Wang et al. [175] compared the effects of different thawing methods on the microstructure of porcine *longissimus lumborum*, as shown in Figure 7. The intermuscular gap of the ultrasound thawed sample (0.34 mm^2^) was higher than that of the vacuum thawed sample (0.12 mm^2^), but significantly smaller than that of the microwave thawed sample (2.42) and the water immersed thawed sample (1.23), indicating that ultrasound thawing caused less damage to meat tissue. Shi et al. [165] reported that synchronous dual-frequency ultrasound significantly accelerated the thawing of goose meat while maximally preserving its quality by reducing water loss and structural damage.

Wang et al. [176] found that ultrasonic treatment (400 W, 45 kHz) during the thawing stage exhibited the most pronounced improvement in WHC compared to other freezing stages, achieving a WHC value of up to 0.78 on a scale of 0 to 1 (with higher values indicating better water-holding capacity), as determined by the freeze–thaw cycle method described in that study. In addition, ultrasound-assisted thawing helps to the preserve protein functional properties, such as gelation behavior. Wang et al. [177] investigated the effects of sweeping-frequency and fixed-frequency ultrasound-assisted thawing on the gel properties of myofibrillar proteins from small yellow croaker. The results indicated that proteins subjected to sweeping-frequency ultrasound thawing formed gels with superior elasticity and lower apparent viscosity, with water distribution more closely resembling that of fresh samples. Beyond textural preservation, ultrasound-assisted thawing of frozen duck meat at 400 W effectively reduced the formation of off-flavor compounds by inhibiting lipid oxidation and free amino acid degradation compared with non-ultrasound treated samples [164,178].

### 3.8. Ultrasonic Application in Other Meat Processing Procedures

#### 3.8.1. Drying

Drying is one of the oldest and most extensively used meat preservation method, involving heat and mass transfer processes. It is often used in the manufacture of fermented sausages, meat powders, and dry-cured hams [179]. Ultrasound-assisted drying has attracted increasing attention in the processing of various food materials due to its ability to significantly reduce drying time and energy consumption, as demonstrated in plant-based products [180]. For example, in vegetable processing, tri-frequency ultrasound-assisted blanching has been shown to inactivate enzymes more efficiently than conventional hot-water blanching, resulting in a significant reduction in subsequent drying time [181]. Beyond accelerating drying kinetics, ultrasound-assisted drying has been reported to improve the retention of bioactive compounds and nutritional components in plant-derived materials [182,183], leading to dehydrated products with higher nutritional quality [184,185].

Ultrasound-assisted drying is frequently combined with other drying technologies to achieve synergistic effects. Early studies demonstrated that ultrasound-assisted vacuum drying significantly decreased drying time and energy consumption in meat products [186]. When applied to minced meat, ultrasound-assisted vacuum drying not only markedly reduced drying time but also produced products with improved rehydration capacity, microstructure, and oxidative stability than conventional vacuum drying and freeze-drying [187]. For highly viscous materials, like honey, ultrasound-assisted vacuum drying has also been proven to significantly shorten drying duration [188]. In addition to vacuum-based systems, ultrasound has been integrated with microwave drying, supercritical CO_2_ drying, infrared drying, catalytic infrared drying, and microwave-hot air rolling drying to further enhance drying efficiency [189,190]. Gao et al. [190] innovatively combined contact ultrasound and infrared radiation with conventional hot-air drying. This synergistic approach reduced drying time, improved color and tenderness, and accelerated the conversion of proteins and lipids into flavor compounds by regulating enzyme activity and chemical reactions, thereby comprehensively enhancing the quality of air-dried beef.

The underlying principles of ultrasound-assisted drying are primarily related to cavitation-induced disruption of the moisture boundary layer and the formation of microchannels within tissues, which accelerate moisture migration. In addition, microjets enhance internal convective mass transfer, resulting in more uniform drying. The mild thermal effects of ultrasound enable drying at lower temperatures. Collectively, these processes work together to reduce drying time while better preserving the texture, color, and nutritional value of meat products.

#### 3.8.2. Extraction

Ultrasound technology has been widely applied for the extraction of bioactive compounds, flavor substances, and functional proteins from various food matrices, including plant materials [191,192] as well as from meat by-products [193]. The primary mechanism involves acoustic cavitation, which disrupts muscle tissues and cell membranes, enhances solvent penetration into the matrix, accelerates solute diffusion, and ultimately improves extraction efficiency, as demonstrated in both plant and animal tissues [194,195]. Numerous studies have demonstrated that ultrasound-assisted extraction recovers higher yields of water-soluble proteins, amino acids, peptides, and flavor precursors under relatively mild conditions in various food systems [196,197], while simultaneously reducing extraction time and energy consumption compared with traditional methods [198].

For example, ultrasound-assisted aqueous extraction or enzymatic hydrolysis significantly increased the yields of sarcoplasmic proteins and myofibrillar protein-derived peptides while maintaining favorable functional properties [199,200]. Collagen-rich bovine digital flexor tendons are underutilized by-product in the meat industry. Studies have demonstrated that ultrasound and pepsin treatment markedly enhances collagen extraction efficiency while keeping the structural integrity and quality of the isolated collagen [193], which is consistent with the findings of Schmidt et al. [201]. Furthermore, ultrasound has been shown to regulate collagen self-assembly, gel structure, and digestibility, highlighting its potential in the development of collagen-based products [202].

By enhancing solvent-solid contact and accelerating molecular diffusion, ultrasound can also improves the extraction efficiency of volatile aroma compounds and antioxidant components from plant materials [203], as well as from marine by-products [204]. However, excessive ultrasound intensity may adversely affect product quality. For instance, ultrasound-assisted solvent extraction significantly increased lipid and carotenoid yields from Pacific white shrimp cephalothorax, but also led to pronounced lipid oxidation and hydrolysis [204]. Similar observations were reported by Sinthusamran et al. [205], who found that ultrasound-assisted extraction effectively recovered DHA/EPA and astaxanthin, while simultaneously promoting oxidative and hydrolytic degradation.

## 4. Disadvantages of Ultrasound-Assisted Meat Processing

Although ultrasound-assisted technologies are generally regarded as beneficial in meat processing, their potential adverse effects should not be overlooked. The efficacy of ultrasound application is highly dependent on the selection of appropriate ultrasonic parameters, including frequency, power intensity, treatment duration, and system configuration, with optimal conditions varying substantially depending on equipment type, animal species, meat cut, and sample geometry [206]. This variability poses significant challenges for process standardization and industrial-scale implementation [9].

A increasing amount of data suggests that excessively high ultrasonic intensity or prolonged treatment duration may induce undesirable effects. Lipid oxidation is recognized as one of the most serious adverse effects associated with ultrasound treatment. The collapse of cavitation bubbles causes localized severe circumstances and large quantities of reactive oxygen species (ROS), such as hydroxyl radicals (•OH), which can directly attack unsaturated fatty acids and initiate or accelerate lipid autoxidation chain reactions. Lipid peroxidation is often accompanied by off-flavor formation and the oxidative denaturation of proteins, reducing the functional and nutritional properties of meat products [207]. For example, during grass carp freezing, excessive ultrasonic power significantly promoted lipid oxidation, which in turn induced protein cross-linking and exacerbated oxidative deterioration [208]. Similarly, in ultrasound-assisted meatball frying, increased ultrasonic power has been reported to accelerate oxidative reactions while suppressing the formation of desirable flavor compounds [76].

In addition to promoting lipid oxidation, excessive ultrasonication may induce excessive unfolding of protein molecules, leading to the exposure of hydrophobic groups. This process facilitates non-specific intermolecular aggregation and the development of insoluble protein aggregates, which can severely impair protein functionality, including solubility, emulsifying capacity, and gelation properties [209,210]. In surimi systems, for example, excessive ultrasound radiation weakened gel strength, resulting in loose product structure and reduced elasticity, while concurrently decreasing WHC and increasing moisture loss [211].

Furthermore, localized or overall temperature increases induced by HIU may alter the appearance, color, and flavor of meat products, severely affecting sensory attributes. From an industrial perspective, ultrasound processing is often applied to bulk materials, where densely stacked meat pieces can hinder effective ultrasound propagation. Uneven energy distribution within the product matrix may cause uneven treatment intensity, resulting in non-uniform quality attributes in ultrasound-treated meat products.

## 5. Conclusions and Future Trends

Ultrasound technology, as an emerging non-thermal physical processing approach, has demonstrated substantial application potential and multifaceted benefits in meat processing. This review systematically summarizes recent advances in ultrasound-assisted meat processing, covering a broad range of applications including tenderization, cooking, brining, sterilization, fermentation, freezing, thawing, drying, and extracting value-added components from meat by-products.

Overall, ultrasound enhances meat tenderness by disrupting muscle integrity and modifying collagen structure, while also accelerating conventional cooking processes and offering an energy-efficient and time-saving alternative for rapid meat cooking. In curing applications, ultrasound effectively shortens brining time and promotes more uniform salt diffusion. Furthermore, the ability of ultrasound to modulate microbial activity highlights its promising role in both microbial inactivation and controlled meat fermentation. In addition, ultrasound-assisted freezing and thawing significantly reduce processing time and quality deterioration associated with conventional freeze–thaw cycles, while ultrasound-assisted drying and extraction further improve dehydration efficiency and the yield of bioactive compounds. Importantly, ultrasound not only accelerates processing operations but also helps preserve or enhance key physicochemical and functional properties of meat products, including enhanced tenderness, WHC, and color stability, promoted flavor development, and reduced cooking loss. These advantages collectively position ultrasound as a versatile and powerful tool for process intensification in modern meat processing.

Despite its promise, several limitations currently hinder the industrial-scale adoption of ultrasound technology. Therefore, future research should delve into the molecular mechanisms elucidation that regulate the interaction between ultrasound and complex meat matrices (proteins, fats, and water), which can support salt reduction to align with health-conscious consumption trends and precisely modifying proteins for developing functional and restructured meat products. Additionally, synergistic strategies, combining ultrasound with technologies like high pressure, electrical pulses, and antioxidants to maximize their technical advantages, should be actively explored. A key focus should be addressing the issue of uniform sound field distribution in large processing tanks to ensure consistent treatment outcomes, ultimately achieving comprehensive improvements in meat product quality.

## Figures and Tables

**Table 1:** Classification of ultrasound based on different criteria.

Classification Criterion	Category	Typical Parameters	Primary Application in Food Processing
Power density	Low-intensity	<1 W/cm^2^	Non-destructive analysis, monitoring
	High-intensity (power ultrasound)	10–1000 W/cm^2^	Process intensification (e.g., tenderization, sterilization, freezing)
Power-frequency relationship	High-power low-frequency	20–100 kHz	Cavitation-induced physical/chemical effects
	Medium-power intermediate-frequency	100 kHz–1 MHz	Limited cavitation, some mass transfer
	Low-power high-frequency	1–100 MHz	Analytical/diagnostic uses
Frequency modulation	Swept frequency	Varies	More uniform energy distribution
	Fixed frequency	Constant	Conventional ultrasound systems
Emission mode	Pulsed	On/off cycles	Reduced heating, controlled cavitation
	Continuous	Constant emission	Higher energy input, continuous effects

**Table 2:** Summary of ultrasound-assisted tenderization in meat and meat products.

Material	Treatment Methods	Optimal Parameters	Main Results	References
Chicken gizzards	US	500 W, 30 kHz, 3 s/3 s, 30 min	Decreasing the shear force and muscle fiber diameter by 27.1% and 26.2%, increasing the myofibril fragmentation index by 238.1%, and lowering hydroxylysine pyridinoline and lysine pyridinoline by 23.1% and 40.5%, respectively.	[47]
Yak meat	US + Lactic acid + Papain	350 W, 45 kHz, 30 min; 0.03% lactic acid, and 200 U/g Papain	Exhibiting a 62.16% reduction in cutting force, a 31.25% decrease in cooking loss, and a 4.3-fold increase in the myofibrillar rupture index.	[48]
Old chicken breast meat	US + Potassium alginate (PA) (UPA)	300 W, 20 kHz, 15.6 W/cm^2^, 5 min; (PA; 0.2–1.0%, 25 min)	UPA–0.4% group exhibiting the lowest moisture loss (drip loss: 1.29 ± 0.09 g/kg and cooking loss: 16.53 ± 0.20 g/kg), and shear force (12.67 ± 0.52 N), respectively.	[49]
Beef	US	37 kHz, 90 W/cm^2^ 25 min (bath); or 24 kHz, 400 W, 50 min (probe)	Decreasing the water holding capacity and shear force by 3.1–5% and 0.59–0.72 kgf, favoring the muscle tenderization after storage, and significantly increasing the muscle lightness, respectively.	[50]
		45 kHz, 11 W/cm^2^, 20 min, 4.7–6 °C	Increasing the luminosity and yellowness (b*), and decreasing the shear force of meat, showing a tenderizing effect.	[51]
Spent-hen breast meat	US + Papain	300 W, 40 kHz, 30 °C, <20 min	Significantly reducing shear force, and increasing water holding capacity (*p* < 0.05).	[52]

**Table 3:** Summary of the antimicrobial action of ultrasound on meat and meat products.

Material	Treatment Methods	US Parameters	Main Results	References
Fermented pork jerky	US	480 W, 30 kHz, 30 min	No coliform bacteria, *Salmonella*, or *Shigella* were detected during the storage. *S*. *aureus* was first observed on day 18.	[109]
Chicken breasts	US	40 kHz, 9.6 W/cm^2^, 50 min	The number of *S. aureus* decreased significantly after 7 d of refrigeration.	[108]
Cooked smoked lamb products	US	35 kHz, 2 min; 26 kHz, 1 min	*C. albicans*, *E. coli*, *B. subbillis*, and *S. aureus* was reduced by 33.3%, 43.8%, 46.8%, and 80.6% by US (35 kHz, 2 min). US (26 kHz, 1 min) reduced them by 50%, 64.6%, 89.1%, and 86.8%, respectively.	[110]
Semitendinosus beef muscle	US	40 kHz, 11 W/cm^2^	Significantly reducing the counts of mesophilic bacteria, psychrophilic bacteria, *Staphylococcus* spp., and coliform bacteria.	[111]
Pork meat	US + heating	20/40/60 kHz, 70 °C heating, 5 min	Reduction in *B*. *cereus* spores at 4.16 log CFU/mL	[112]
Tuna fish	US + slightly acidic electrolyzed water (SAEW)	55 mg/mL SAEW + 280 W US	Demonstrating the strong antibacterial effect during storage, with a total viable count (TVC) of 3.11 ± 0.01 log CFU/g.	[113]
Chicken meat	US + Peppermint essential oil	37 kHz, 600 W, 2/4/6 min	During refrigeration (12 d, 4 °C), the treated group exhibited the total plate count, *Salmonella*, coliform, and *E. coli* of 1.53–3.76, 1.21–1.99, 1.08–1.48, and 1.95–2.99 CFU/g, significantly lower than untreated group of 2.4–7.71, 3.56–5.61, 1.87–4.41, and 4.47–7.23 CFU/g, respectively.	[114]
	US	20 kHz, 27.6 W/cm^2^; 40 kHz, 10.3 W/cm^2^; 850 kHz, 24.1 W/cm^2^	Microbial counts in samples treated at 20 kHz (3.7 ± 0.4 and 8 ± 0.6 log CFU/g) were significantly lower than the control and the groups treated at 40 kHz and 850 kHz.	[115]
	US + sodium hypochlorite (SH)	25 kHz, 6 kw, 25 min	US + SH and 2 × (US + SH) reduced TVC, *Enterobacteriaceae*, and psychrophilic bacteria on the surface at day 0, while preserving sensory quality during 5 d of chilled storage. 2 × (US + SH) achieved reduction in TVC (4.72 log CFU/g), *Enterobacteriaceae* (2.83 log CFU/g), and psychrophilic bacteria (4.89 log CFU/g), inhibiting the growth of *Acinetobacter*, *Aeromonas*, *Shewanella*, and *Pseudomonas*, extending shelf life by more than 2 d.	[116]

**Table 4:** Summary of ultrasound-assisted meat freezing.

Material	Treatment Methods	US Parameters	Main Results	References
Chicken breast	US in an air-forced cooling tunnel	37% net sonication time; 40 kHz; 50 W, −13 to −22 °C	Reducing freezing time by 11%. No significant differences between US-assisted frozen samples and the control in WHC and cooking loss.	[151]
	Ultrasound-assisted immersion freezing (UIF)	165 W	UIF produced smaller ice crystals than immersion freezing (IF) and air freezing (AF). It significantly reduced thawing and cooking losses, color deterioration, and lipid oxidation levels during frozen storage (*p* < 0.05). UF minimized water migration throughout storage (*p* < 0.05).	[149]
*Sciaenops ocellatus*	UIF	150, 200, and 250W, 25 kHz	UIF (200 W) increased freezing rate by 712.81% and shortened freezing time by >83% compared with AF. UIF (200 W) group had higher protein stability after 90 days of storage.	[152]
Macrobrachium rosenbergii	Multi-frequency ultrasound-assisted immersion freezing (MUIF)	MUIF (20 + 40 kHz), (20 + 60 kHz), (40 + 60 kHz), and (20 + 40 + 60 kHz), 180 W	The average diameter of ice crystals in IF is 28 μm, whereas in MUIF-20 + 40 + 60 it is only 8 μm. MUIF mitigates oxidative degradation of lipids and proteins.	[153]
Beef	UIF	0–400 W, 24 kHz	UIF (125 W, 50% duty cycle) significantly reduced freezing time, thawing and cooking losses, and improved color quality of the samples.	[154]
Sea bass	UIF	320 W, 45 kHz, −20 ± 0.5 °C and −40 ± 0.5 °C	UIF (at −40 °C) lowered ice crystals, maintained stable proteins during frozen storage, and reduced thawing and cooking losses.	[155]

**Table 5:** Summary of ultrasound-assisted thawing of meat.

Material	Treatment Methods	US Parameters	Main Results	References
White yak meat	US-assisted thawing (UAT)	0, 200, 400, and 600 W, 20 kHz	Shortening thawing time by 30.95–64.28%; reducing thawing loss, cooking loss, L* and b* values, and pH (*p* < 0.05), whereas increasing a* value and cutting force, free amino acids, minerals, water-soluble vitamins, and volatile compounds (*p* < 0.05).	[167]
Lamb	UAT	350 W, 40 kHz	Improving the water retention capacity, preserving color, and effectively inhibiting protein oxidation (*p* < 0.05).	[168]
Duck meat	UAT	200, 400, and 600 W, 40 kHz	Shortening thawing time by 30.96–55.05% with power (200 to 600W), reducing thawing loss, pH, L*, b*, shear force, and pressure water loss, and increasing a*, color, tenderness, and WHC (*p* < 0.05) by UAT (400 W), and reducing the off-flavor.	[164]
Chicken breast	UAT	200, 300, 400, and 500 W, 15 ± 0.5 °C	UAT (300 W) decreasing thawing time by 57% compared to air thawing, and reducing the damage to myofibrillar protein structure.	[166]
Goose meat	Simultaneous dual-frequency US (SDU)	Combining 20, 25, 28, 40, 50 kHz in pairs, 25 ± 1 °C, 50 W/L	Shortening thawing time by 17.76–36.06% over running water thawing; achieving the lowest thawing loss (13.36%) and surface hydrophobicity (67.76 μg) by SDU (28 + 50 kHz).	[165]
Pork	US (mono-, dual- or tri-frequency sequential and simultaneous mode)	20, 35, 50 kHz; 20/35, 20/50, 35/50 kHz; 20/35/50, 20/50/35 kHz; 20 + 35, 20 + 50, 35 + 50, and 20 + 35 + 50 kHz, 40 W/L	Reducing thawing time by 26.72–64.99%, inhibiting lipid oxidation; UAT (20/50/35 kHz) achieving superior water retention (lower thawing and cooking losses) and physicochemical quality (lowest hardness, highest tenderness, and reduced TBARS values), with microstructure most resembling fresh meat.	[169]

## Data Availability

No new data were created or analyzed in this study.
